# Development and validation of multiparametric MRI-based nomogram for predicting occult metastasis risk in early tongue squamous cell carcinoma

**DOI:** 10.1186/s12885-021-08135-6

**Published:** 2021-04-15

**Authors:** Pensiri Saenthaveesuk, Le Yang, Bin Zeng, Meng Xu, Simon Young, Guiqing Liao, Yujie Liang

**Affiliations:** 1 Department of Oral and Maxillofacial Surgery, Guanghua School of Stomatology, Guangdong Provincial Key Laboratory of Stomatology, Sun Yat-sen University, 56 West Lingyuan Road, Guangzhou, 510055 Guangdong China; 2 Department of Oral and Maxillofacial Surgery, Faculty of Dentistry, Khon Kaen University, Khon Kaen, Thailand; 3 Department of Oral Pathology, Guanghua School of Stomatology, Guangdong Provincial Key Laboratory of Stomatology, Sun Yat-sen University, Guangzhou, Guangdong China; 4 Department of Oral and Maxillofacial Surgery, The University of Texas Health Science Center at Houston, School of Dentistry, Houston, TX USA

**Keywords:** Magnetic resonance imaging, Neoplasm invasion, Tumor thickness, Tongue cancer, Squamous cell carcinoma, Neoplasm metastasis, Nomograms, Cohort studies

## Abstract

**Background:**

Nomograms are currently used in predicting individualized outcomes in clinical oncology of several cancers. However, nomograms for evaluating occult nodal metastasis of patients with squamous cell carcinoma of lateral tongue (SCCLT) have not been widely investigated for their functionality. This retrospective cohort study was designed to address this question.

**Methods:**

This study was divided into primary and validation cohorts. The primary cohort comprised 120 patients diagnosed between 2012 and 2017, whereas the validation cohort included 41 patients diagnosed thereafter. The diagnostic value of multiparametric MRI, including radiologic tumor thickness threshold (rTTT) in three-dimensions, paralingual distance, and sublingual distance were investigated. A nomogram was developed based on stepwise logistic regression of potential predictors associated with nodal metastasis in the primary cohort and then tested for predictive accuracy in the validation cohort using area under the curve (AUC) and goodness-of-fit tests.

**Results:**

Multivariate analysis, tumor size (odd ratio [OR] 15.175, 95% confidence interval [CI] 1.436–160.329, *P* = 0.024), rTTT (OR 11.528, 95% CI 2.483–53.530, *P* = 0.002), paralingual distance (OR 11.976, 95% CI 1.981–72.413, *P* = 0.005), and tumor location (OR 6.311, 95% CI 1.514–26.304, *P* = 0.011) were included in the nomogram to predict the likelihood of having cervical metastasis. A nomogram cutoff value of 210 points (sensitivity 93.8%, specificity 87.5%) was significantly different to classify the patients metastasis risk group (*P* < 0.001). Nomogram showed predictive accuracy with AUC 0.881 (95% CI 0.779–0.983, *P* < 0.001) and good calibration after the validation.

**Conclusions:**

A preoperative nomogram incorporating multiparametric MRI demonstrated good prediction and performed adequately in our study. Three-dimensional assessment of occult metastasis risk value obtained from this nomogram can assist in preoperative decision making for individual patients with early-stage SCCLT. The probability of nodal metastasis tended to be greater than 20% in patients with high metastasis risk or nomogram total score > 210 points.

## Background

Management of patients with clinically node-negative (cN0) early-stage oral cancer remains controversial [1–6]. Early stage (T1 and T2) oral tongue cancer has a strong tendency to metastasize to regional lymph nodes subclinically, with metastasis rates between 23 and 40% [7–10]. There are various factors that influence prognosis of survivability in oral cancer patients, and pathologic depth of invasion (pDOI) is currently the most accurate predictor of occult metastasis. pDOI should be used to guide decision-making according to the newly updated American Joint Committee on Cancer (AJCC), 8th Edition. pDOI is useful for deciding postoperative adjuvant treatment and conducting follow-up, but it cannot be accurately assessed in the pretreatment evaluation.

There are several imaging methods available for preoperative evaluation. Recent studies suggest that the high resolution magnetic resonance imaging (MRI) reliably depicts soft tissue tumor extension, and can be used to predict cervical lymph node metastasis by assessing primary tumor thickness [11–13]. Previous studies have reported that MRI-assessed tumor thickness is correlated strongly and directly with pathologic thickness [14–22]. Various MRI techniques have been proposed to assess the tumor thickness of tongue tumors. There are some controversies regarding the significance of a precise clinically optimal cutoff value of the MRI-assessed tumor thickness. A borderline MRI-assessed tumor thickness cutoff value for cervical lymph node involvement has a broad range varying from 4 mm to 14.5 mm. The optimal cutoff value has not been established and there are no standardized techniques to properly measure MRI-assessed tumor thickness.

Nomograms are currently the most reliable discriminating tools for predicting individualized outcomes in clinical oncology to develop a treatment plan and follow-up strategies in several cancers [23–25]. Previous studies have reported nomograms can predict long-term survival rate and cervical metastasis in head and neck cancer patients [26–30]. To date, a nomogram for predicting the likelihood of cervical lymph node metastasis pre-operatively of individual patients with early-stage of squamous cell carcinoma of lateral tongue (SCCLT) has not been widely investigated and no study has verified functionality of a MRI-based nomogram.

Solid cancer cells typically spread in different planes to invade surrounding structures as three-dimensional features. Kwon et al. [21] conducted the three-dimensional (3D) measurement of MRI-assessed tumor thickness. They reported MRI-assessed tumor thickness in 3D was a potential predictive indicator to evaluate occult cervical metastasis. Likewise, Okura et al. [16] reported that tumor thickness and the distance to the paralingual space assessed in the MRI coronal view had a predictive ability for nodal metastasis in SCCLT. It is presumed that cancer cell metastasis occurrs through lymphovascular drainage inferior to the intrinsic tongue muscles to the cervical lymph node.

The present study is designed with two cohort series and aimed to (1) evaluate if the identified optimal cutoff value of multiparametric MRI in three-dimensions can predict the presence of cervical lymph node involvement, (2) establish an accurate nomogram based on MRI for preoperative assessment of the individual patient for occult metastasis risk and (3) determine the validity of a proposed MRI-based nomogram.

## Methods

### Patient selection

#### Primary and validation cohorts

This study was approved by the Institutional Review Board of Hospital of Stomatology, Sun Yat-sen University (SYSU). The eligibility criteria included patients from the Hospital of Stomatology, SYSU diagnosed with untreated cN0 early stage SCCLT and a MRI obtained from the medical diagnosis center, Sixth Affiliated Hospital of SYSU, between January 2012 and November 2017. Exclusion criteria in this study was defined as patients with incomplete medical record data, clinical metastasis, MRI conducted from other facilities, suspected lymph node metastasis appreciated from MRI, exophytic and endophytic tumors, and prior chemotherapy, radiation therapy, and/or tumor excision. A retrospective study was performed and included chart review of 120 patients at Hospital of Stomatology, SYSU in the primary group. Sample size *n* = 120 was determined after a literature review of similar studies, most of which had less than 100 patients were evaluated [14–22]. This sample size was approved by the Insititutional Review Board. To examine the application of the nomogram, external validation was performed using a separate cohort of 41 unique patients that presented in the same institution between December 2017 and August 2018 that had complete data to score all the variables in the nomogram. No data from the validation cohort were used to derive the nomogram, and no data from the primary cohort were used to validate the nomogram.

All patients received surgery as definitive treatment, which included patients who received primary resection with elective neck dissection (END) and adjuvant therapy as indicated. Adjuvant radiotherapy was considered for patients with positive lymph nodes, perineural invasion (PNI), and lymphovascular invasion (LVI). Concurrent chemoRT was indicated in patients with extranodal extension or positive surgical margins. All patients who were followed up for at least 12 months were included in our study. Patients were evaluated every 1–3 months in the first year, every 2–6 months in the second year, every 4–8 months in years 3–5, and every 12 months thereafter.

### MRI and pathologic parameters measurement

**Fig. 1:**
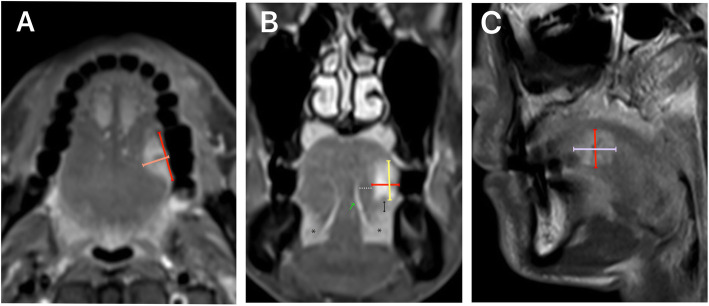
T1W1 MRI showed measurement of MRI-assessed tumor thickness in three dimensions. All reference lines (red line) were defined as the longest tumor diameter in each direction. **a**. Axial tumor thickness (aTT) (orange line), **b**. Coronal tumor thickness (cTT) (yellow line). **c**. Sagittal tumor thickness (sTT) (purple line). In the coronal view, a thin high signal intensity (green arrow line) was used to identify the deep lingual artery and inferior lateral area of high signal intensity (asterisk) was indicated the sublingual space. Paralingual distance was defined as the distance between the lateral aspect next to the deep lingual artery and medial border of the tumor (dotted line). Sublingual distance was determined by measuring the vertical distance between the inferior border of tumor and the sublingual space (black arrow line)

MRI was performed using the 1.5-T system Siemens MR with the following parameters: field of view: 215.3 mm, acquisition matrix: 156 × 256, and slice thickness: 3 mm. Preoperative multiparametric MRI were measured in three-dimensions (axial, coronal, and sagittal views) with spin-echo sequences on contrast-enhanced, fat-suppressed T1WI (TR/TE:574 ms/9.9 ms) were obtained. A single-blinded reviewer (a specialist in the field of head and neck radiology) provided independent interpretation without knowledge of the clinicopathologic findings. MRI assessment of tumor thickness was initially determined by identifying the plane of tumor thickness with a reference line drawn as the longest tumor diameter in each plane. The length was measured from the point of maximal tumor projection perpendicular to the reference line to the deepest infiltration point, represented as MRI-assessed tumor thickness. The same measurement method was used to determine MRI-assessed tumor thickness in each plane. Consequently, axial tumor thickness (aTT), coronal tumor thickness (cTT) and sagittal tumor thickness (sTT) were defined as MRI-assessed tumor thickness in the axial, coronal, and sagittal view, respectively as described in Fig. 1. To improve the accuracy of MRI-assessed tumor thickness in detecting of cervical metastasis, patients were defined as meeting a “radiologic tumor thickness threshold (rTTT)” with a predictor representing MRI-assessed tumor thickness in three-dimensions. The values obtained from aTT, cTT and sTT measurements were evaluated for each patient and could be categorized as “minimal” or “significant” rTTT, depending on how many MRI-assessed tumor thicknesses exceed their cutoff values. Minimal rTTT was defined as only one or fewer orientations of MRI thickness (either aTT, cTT or sTT) found to be greater than its defined cutoff value. Significant rTTT was defined as at least two orientations of MRI thickness (either aTT, cTT or sTT) exceeding their respective cutoff values.

Paralingual distance (PLD) and sublingual distance (SLD) as described by Okura et al. [16] were determinmed in coronal T1-weighted MRI and are illustrated in Fig. 1b. The inferior lateral aspect of the tongue demonstrating high signal intensity represented the sublingual space. The SLD was determined by measuring the vertical distance between the inferior border of the tumor and the sublingual space. The paralingual space was defined as the thin high signal intensity extending from the medial aspect of sublingual space along the genioglossus or hyoglossus muscle (low signal intensity area) to the deep lingual artery. PLD was defined as the distance between the medial border of the tumor and the paralingual space. For lesions in which tumor extension occurred beyond the deep lingual artery and the sublingual space, PLD and SLD were determined to be 0 mm.

Surgical specimens of the tongue were fixed in 20% formalin, embedded in paraffin, and subsequently stained with hematoxylin and eosin for examination through light microscopy. This study included only flat tumors in which the mucosal plane was at the same level as the tumor. The pathologic depth of invasion and tumor thickness could then be measured identically (pDOI = pTT) [13]. An oral pathologist, who was unaware of the MRI findings, oriented the specimen and routinely performed measurement on tumor depth of invasion. Only pathologic thickness in the axial view was available in our institution during the study period. Postoperative pathological parameters of cell-differentiation, presence of lymphovascular invasion (LVI), muscular invasion and perineural invasion (PNI) were also investigated.

### Statistical analysis

All statistical analyses were conducted using SPSS (version 21; Chicago, IL, USA) and R software (version 3.6). Descriptive variables of patient characteristics and multivariable MRI were analyzed. Receiver operating characteristic (ROC) curve analysis was conducted to identify the optimal cutoff value of each MRI parameter in evaluating cervical metastases. A ROC curve was drawn on the basis of the sensitivity and specificity, and the area under the curve (AUC) was also calculated. Pearson correlation coefficient was used to assess the correlation between MRI and pathological thickness in the axial plane. Diagnostic accuracy, including sensitivity, specificity, positive predictive value (PPV), negative predictive value (NPV), positive likelihood ratio (+LR), and negative likelihood ratio (−LR), were evaluated in correct identification of positive and negative neck nodes.

The study population was divided into primary and validation cohorts. A binary logistic regression predictive model was used to construct the nomogram in the primary cohort, and was then used to verify the predictive accuracy in the validation cohort. On multivariate logistic regression, we included multiple variables that had significant associations with occult nodal metastasis in the final nomogram. Nomogram performance was quantified in terms of discrimination and calibration in both cohorts. ROC curve methodology was used to assess the discrimination power and total score cutoff value of the nomogram. The AUC was used to quantify the predictive accuracy of nomogram. Calibration was assessed graphically by plotting the relationship between actual and predicted probabilities by using the Hosmer goodness-of-fit test. Model validation was performed using the bootstrapping method (1000 replications) to quantify our modeling strategy. A value of *P* < 0.05 was considered to indicate statistical significance.

## Results

### Patient characteristics

The characteristics of patients in both cohorts are summarised in Table 1. Of the 120 patients in the primary cohort, 49.2% (59/120) had cN0 T1 tumors, and 50.8% (61/120) had T2 tumors. The treatment modality was recorded for all patients. A total of 120 patients were treated with primary resection and END, 26.7% (32/120) of whom had nodal metastasis (pN+). The mean values of aTT, cTT, sTT, PLD and SLD was 9.89 ± 4.07 mm, 13.44 ± 5.63 mm, 14.35 ± 6.52 mm, 4.38 ± 2.47 mm and 3.80 ± 2.94 mm, respectively. The mean follow-up time was 45 months (18–82 months). Recurrence or metastasis occurred in 15% (18/120): at the primary site in 5% (6/120), in the neck in 4.17% (5/120) and 5.83% (7/120) had distant metastasis in the primary cohort.

**Table 1:** Patient characteristics

Patient characteristics N (%)	Primary Cohort (*N* = 120)	Validation Cohort (*N* = 41)
Age at diagnosis [year]		
• ≤ 60	87 (72.5)	24 (58.5)
• > 60	33 (27.5)	17 (41.5)
Gender		
• Male	71 (59.2)	23 (56.1)
• Female	49 (40.8)	18 (43.9)
Tumor size		
• T1	59 (49.2)	21 (51.2)
• T2	61 (50.8)	20 (48.8)
Tumor Location		
• Anterior/Middle	76 (63.3)	24 (58.5)
• Posterior	44 (36.7)	17 (41.5)
MRI parameters [mm]		
1.MRI-assessed tumor thickness in 3D		
• Tumor thickness in axial view (aTT)		
≤ 8.5 mm	81 (67.5)	25 (61)
> 8.5 mm	39 (32.5)	16 (39)
• Tumor thickness in coronal view (cTT)		
≤ 11 mm	69 (57.5)	11 (26.8)
> 11 mm	51 (42.5)	30 (73.2)
• Tumor thickness in sagittal view (sTT)		
≤ 12 mm	75 (62.5)	19 (46.3)
> 12 mm	45 (37.5)	22 (53.7)
2. Paralingual distance (PLD)		
≤ 5 mm	60 (50)	24 (58.5)
> 5 mm	60 (50)	17 (41.5)
3. Sublingual distance (SLD)		
≤ 4 mm	73 (60.8)	28 (68.3)
> 4 mm	47 (39.2)	13 (31.7)
Pathological node status		
• pN0	88 (73.3)	29 (70.7)
• pN+	32 (26.7)	12 (29.3)
Postoperative pathological thickness*		
• ≤ 5 mm	23 (19.2)	4 (9.8)
• > 5 mm to ≤10 mm	83 (69.2)	31 (75.6)
• > 10 mm	14 (11.7)	6 (14.6)
Cell-differentiation		
• Well	61 (50.8)	26 (63.4)
• Moderate/Poor	59 (49.2)	15 (36.6)

* This study included only flat tumors in which the mucosal plane was at the same level as the tumor. Then the pathologic depth of invasion and pathological tumor thickness could be measured identically (pDOI = pTT)

For postoperative pathologic results, the mean value of pathologic thickness was 8.06 ± 3.47 mm. Pearson’s correlation coefficient showed a strong positive correlation between pathologic thickness and MRI thickness in mediolateral direction (aTT) with 0.836 (*P* < 0.001). Furthermore, a significant difference was found in predicting cervical lymph node status when incorporating pDOI according to the AJCC. No occult metastasis was detected in pDOI ≤5 mm, metastasis rate was 25.3% in pDOI > 5 mm to ≤10 mm, and metastasis rate was 78.6% in pDOI > 10 mm (*P* < 0.001). The presence of PNI was also significantly different according to nodal status (*P* = 0.046). In contrast, cancer cell differentiation (*P* = 0.824), presence of LVI (*P* = 0.663) and muscular invasion (*P* = 0.304) were not significantly different for predicting nodal metastasis.

### Identifying cutoff values of MRI parameters to estimate pN+

**Fig. 2:**
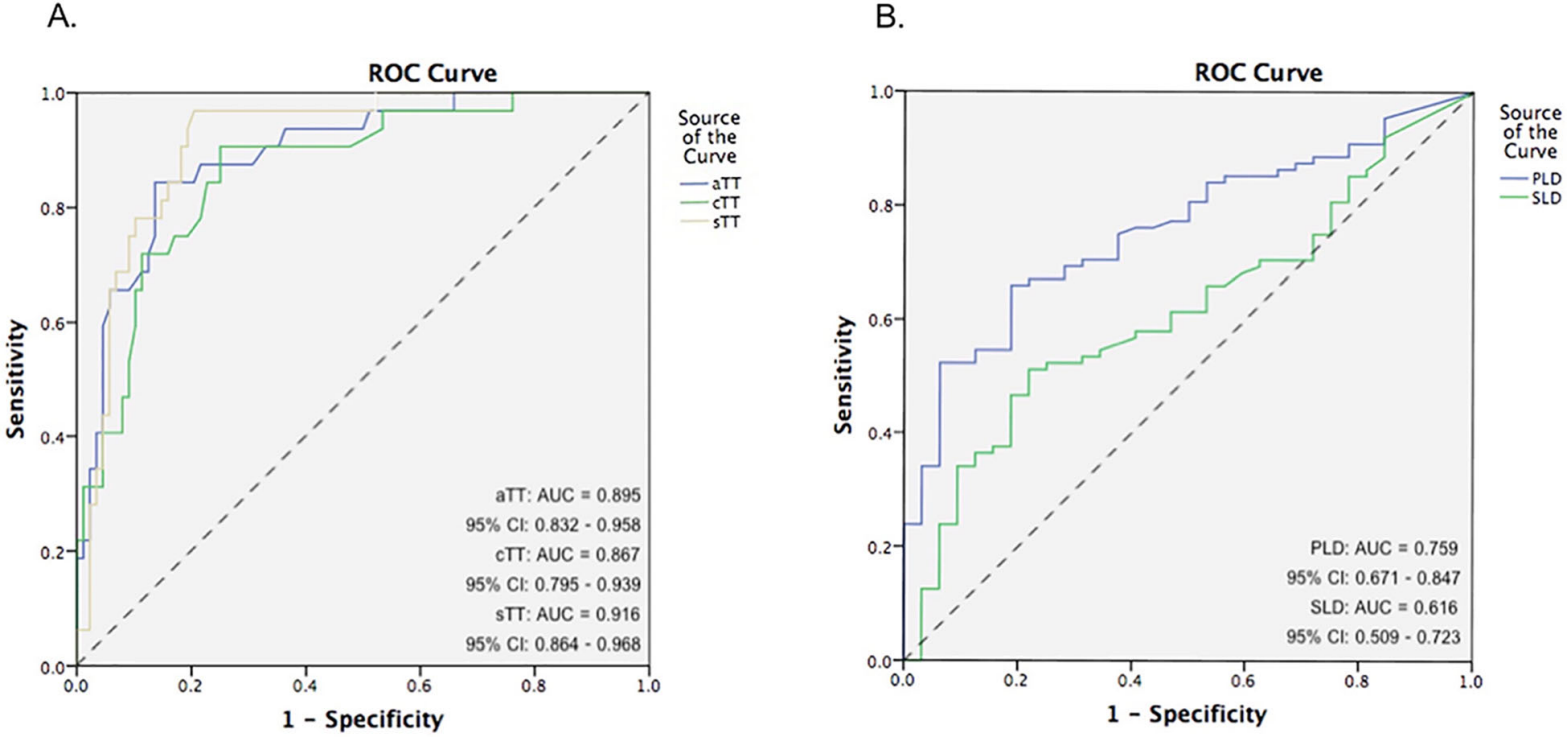
Receiver operation characteristic curve indicated the optimal cutoff value of MRI-multiparameter in evaluating cervical metastasis. **a**. Area under the curve (AUC) of MRI-assessed tumor thickness cutoff values were a statistically significant difference (*P* < 0.05); aTT cutoff value as 8.5 mm was 0.895 (blue line), AUC of cTT cutoff value as 11 mm was 0.867 (green line) and AUC of sTT cutoff value as 12 mm was 0.916 (yellow line). The cutoff for sTT curve being the most significantly different. **b**. Paralingual distance (PLD) and sublingual distance (SLD) cutoff values were 5 mm (AUC = 0.759) and 4 mm (AUC = 0.616), respectively

In this study, occult metastasis risk in early-stage SCCLT was assessed by evaluation of MRI parameters in three-dimensional planes. The effect of cutoff value and its impact on diagnostic accuracy is presented in Table 2. The ROC curve for the proposed cutoff value of predictors to assess subclinical nodal metastases is shown in Fig. 2.

**Table 2:** Predictors diagnostic accuracy using cutoff value to predict presence of pN+ cases

Parameter cutoff value	Sensitivity	Specificity	PPV	NPV	LR +	LR -
aTT 8.5 mm	84.4%	86.4%	69.2%	93.8%	6.00	0.19
cTT 11 mm	87.5%	73.9%	54.9%	94.2%	3.38	0.16
sTT 12 mm	90.6%	81.8%	64.4%	96.0%	5.06	0.11
PLD 5 mm	93.8%	65.9%	50.0%	96.7%	2.76	0.09
SLD 4 mm	81.3%	46.6%	35.6%	87.2%	1.53	0.40

Abbreviations

pN+ = positive neck-node; PPV = positive predictive value; NPV = negative predictive value; LR+ = positive likelihood ratio; LR- = negative likelihood ratio; aTT = MRI tumor thickness in axial view; cTT = MRI tumor thickness in coronal view; sTT = MRI tumor thickness in sagittal view; PLD = paralingual distance and SLD = sublingual distance

ROC curve analysis indicated that the aTT of 8.5 mm, cTT of 11 mm and sTT of 12 mm are the optimal threshold values for predicting cervical lymph node metastases, with the AUC being 0.895, 0.867 and 0.916, respectively. Notably, the predictive value of aTT with an 8.5 mm cutoff value was 84.4% sensitivity, 86.4% specificity, 69.2% PPV, 93.8% NPV, +LR 6.00 and -LR 0.19 (*P* < 0.001). The diagnostic value of sTT with 12 mm cutoff value was 90.6% sensitivity, 81.8% specificity, 64.4% PPV, 96% NPV, +LR 5.06 and -LR 0.11 (*P* < 0.001). Additionally, the incidence of nodal metastasis was higher in patients with significant rTTT than in patients with minimal rTTT (65.1% versus 5.2%, *P* < 0.001). The ROC curves reflected that PLD of 5 mm and SLD of 4 mm are the optimal decision threshold related to nodal metastases, with the AUC being 0.759 and 0.616, respectively.

### Development of multiparametric MRI-derived nomogram for predicting of individualized occult metastasis

A univariate analysis was initially performed to evaluate the association between each variable and lymph node metastases. Besides, rTTT, PLD and SLD determined via MRI assessment, the risk variables of sex, age, tumor size (T1 or T2), pathologic differentiation (well or moderate-poor), and tumor location (anterior-middle or posterior) were evaluated. The probability of lymph node metastasis in each variable was estimated. Then, all variables with a *P* value < 0.05 in the univariate analysis were entered in a multivariate logistic regression analysis model by using a backward condition method. Remaining variables were used to create a nomogram that could be used to predict the probability of cervical lymph node-positive disease in the primary cohort. Multivariate logistic regression analysis demonstrated that tumor size (OR 15.175, 95% CI 1.436–160.329, *P* = 0.024), rTTT (OR 11.528, 95% CI 2.483–53.530, *P* = 0.002), PLD (OR 11.976, 95% CI 1.981–72.413, *P* = 0.005), and tumor location (OR 6.311, 95% CI 1.514–26.304, *P* = 0.011) emerged as significant predictors to indicate the existence of lymph node metastasis of early-stage SCCLT (Table 3).

**Table 3:** Univariate and multivariate analysis to identify potential predictor in predicting regional lymph node metastasis

		Univariate analysis		Multivariate analysis	
		OR (95% CI)	*P* Value	OR (95% CI)	*P* Value
Age	≤ 60	Reference 0.433 (0.137–1.376)	0.209	–	–
	> 60				
Gender	Male	Reference 0.957 (0.390–2.351)	1.000	–	–
	Female				
Tumor size	T1	Reference 59.933 (7.797–460.704)	0.000	Reference 15.175 (1.436–160.329)	0.024
	T2				
Tumor location	Anterior/ Middle	Reference 5.395 (2.259–12.888)	0.003	Reference 6.311 (1.514–26.304)	0.011
	Posterior				
rTTT^*^	Minimal rTTT	Reference 31.067 (10.407–111.518)	0.000	Reference 11.528 (2.483–53.530)	0.002
	Significant rTTT				
PLD	> 5 mm	Reference 29.000 (6.486–129.668)	0.000	Reference 11.976 (1.981–72.413)	0.007
	≤ 5 mm				
SLD	> 4 mm	Reference 3.780 (1.417–10.088)	0.006	–	–
	≤ 4 mm				
Cell-differentiation	Well	Reference 1.241 (0.552–2.792)	0.681	–	–
	Moderate/ Poor				

Abbreviations: OR = odds ratio, CI = confidence interval, PLD = Paralingual Distance, SLD = Sublingual Distance

^*^ rTTT (radiologic tumor thickness threshold) was categorized into two groups: “minimal” and “significant” rTTT, depending on how many MRI-assessed tumor thicknesses exceed their cutoff values. Minimal rTTT was defined as only one orientation of MRI thickness (either aTT, cTT or sTT) found to be greater than its defined cutoff value or no MRI thicknesses exceeding their cutoff values, whereas significant rTTT was defined as at least two orientations of MRI thickness (either aTT, cTT or sTT) exceeding their cutoff values

**Fig. 3:**
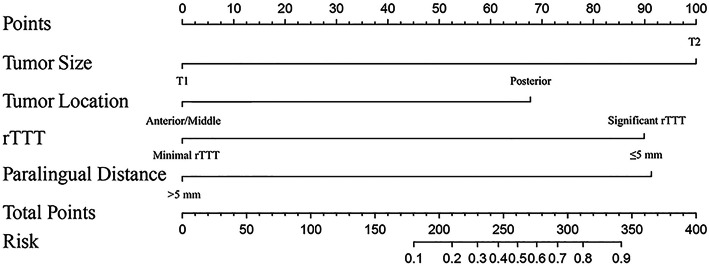
Preoperative nomogram incorporating multiparameter MRI for individualized occult metastasis risk prediction in early-stage SCCLT. Summary of nomogram consisted of a set of scales in which each scale represents a characteristic of the study population. Each predictor to be considered as a point value based on power predictive ability in assessing occult metastasis risk. The points assigned to each of the four predictors and the total points obtained from four predictors are summed and indicated the probability of the patient likelihood of having cervical lymph node metastasis

Ultimately, four potential predictors were presented in the final nomogram to identify the occult metastasis risk and predict the likelihood of cervical nodal involvement for the individual patient (Fig. 3). The nomogram was characterized by one scale corresponding with each variable, a score scale, a total score scale, and a probability scale. The top row displays the point assignment for each predictor. The metastasis risk predictors are: tumor size, tumor location, rTTT and PLD. Each predictor represents a point value based on the tumor characteristics (row 2–4). The points assigned to each of the four predictors are summed, and the total points are indicated in row 5. The bottom row displays the probability of the patient having cervical lymph node metastasis.

### Nomogram use for classifying patient metastasis risk group in the primary cohort

ROC curve analysis indicated that the optimal cutoff value of nomogram total score of 210 points was significantly different to discriminate the patients likelihood of having cervical lymph node metastasis preoperatively. Patient may be categorized as having a low occult metastasis risk depending upon their total risk score of less than 210 points. Patients with a total risk score of more than 210 points were categorized as high occult metastasis risk. The possibility of metastasis rate of higher than 20% when total score was more than cutoff value of 210 points was presented.

**Fig. 4:**
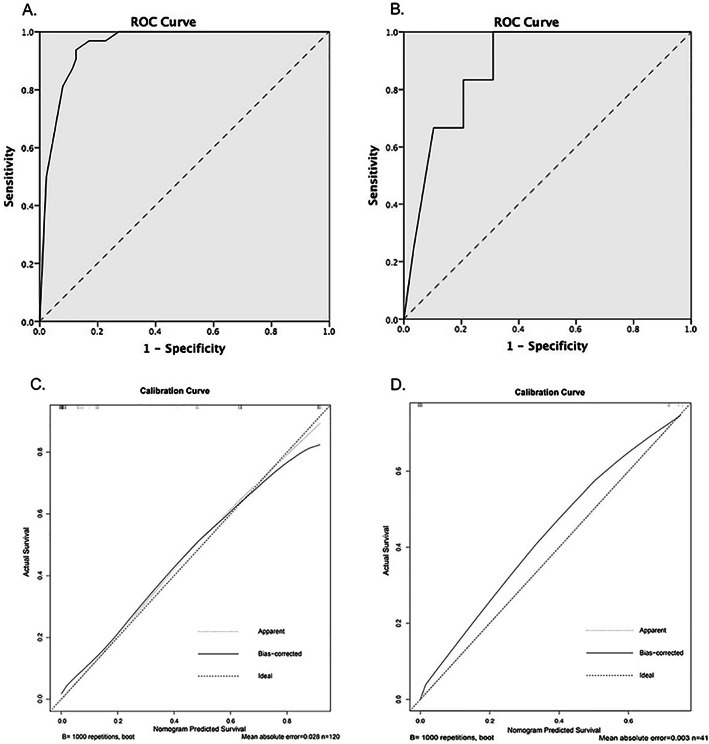
Prediction performance and calibration of the multiparametric MRI-based nomogram in detecting cervical lymph node metastasis. The nomogram cutoff value of 210 points was used to generate the patients into metastasis risk groups in this study. Prediction performance of the multiparametric MRI-based nomogram in the primary (**a**) and validation (**b**) cohorts was measured by the area under the receiver operating characteristic curve. The predictive accuracy to discriminate the patients likelihood of having occult nodal metastasis were 0.952 (95% CI 0.917–0.987, *P*  < 0.001) in the primary cohort and 0.881 (95% CI 0.779–0.983 (*P* < 0.001) in the validation cohort. Calibration curve of the MRI-based nomogram for predicting the risk of occult nodal metastasis in the primary (**c**) and validation (**d**) cohorts of patients with early-stage SCCLT. Calibration curve indicated there had no apparent departure from perfect fit, with good correlation between the prediction and observation in both cohorts.

The MRI-based nomogram demonstrated predictive accuracy for individualized positive cervical lymph nodes, resulting in excellent discrimination power of an AUC 0.952 with 95% CI 0.917–0.987, *P*  < 0.001 in Fig. 4a. The sensitivity, specificity, PPV, and NPV of the nomogram using the cutoff value of 210 points were 93.8, 87.5, 73.2 and 97.5%, respectively (*P* < 0.001). The Hosmer–Lemeshow goodness-of-fit test indicated that the nomogram was well-calibrated and the slope had no apparent departure from perfect fit (*P* < 0.001). The calibration plot showed good agreement between the prediction and metastasis risk observation (Fig. 4c).

### Clinical validation of MRI-based nomogram

Validation cohort analysis was performed for 41 patients who were diagnosed as having T1 tumors 51.20% (21/41), and T2 tumors 48.80% (20/41). All patients were treated with primary resection and END, and 29.3% (12/41) had cervical node metastasis (pN+). The nomogram cutoff value of 210 points generated the patients into metastasis risk groups, 25 patients (60.98%) were defined as low metastasis risk group. A total of 16 patients (39.02%) had a high metastasis risk group. Incidence of nodal metastasis demonstrated a statistically significant difference between the low and high metastasis risk groups (*P* = 0.001). The incidence of nodal metastasis was higher in the high metastasis risk group (62.5%), than in the low metastasis risk group (8.0%).

Validation of the nomogram was conducted both internally and externally, as presented in Fig. 4. In the entire validation cohort, the ROC curve analysis reflected the MRI-based nomogram demonstrated excellent prediction performance with high predictive value of AUC 0.881, 95% CI 0.779–0.983 (*P* < 0.001) in Fig. 4b. Additionally, a diagnostic test was conducted by using a nomogram cutoff value of 210 points. The results showed a sensitivity of 83.3%, specificity of 79.3%, PPV of 62.5%, and NPV of 92.0%. The calibration curves of the nomogram in the validation cohorts were plotted, and the slope was close to 1, indicating that the nomograms were well calibrated in Fig. 4d.

## Discussion

Cancer patients will present with several clinical characteristics that can have either positive or negative influences on predicting the likelihood of the occult metastasis risk. Reviewing these clinical aspects of patient will help to generate an initial metastasis risk profile. Nomograms are widely used in pretreatment evaluation of several cancers because the nomogram is useful scoring and reproducible method. This study demonstrated that the multiparametric MRI-based nomogram is a discrimination tool that can be used to predict individualized metastasis outcome for preoperative evaluation, and high NPV results of the nomogram may help to prevent unnecessary neck surgery and morbidity in patients with early-stage SCCLT. Study of Swawant et al. [29] proposed a nomogram for predicting the risk of neck node metastasis in 103 pathologically node-negative T1-T4 oral cavity carcinoma patients by using a combination of protein expression, ultrastructural alterations and clinicopathological parameters. Likewise, Jiang et al. [30] developed a nomogram to predict the probability of occult cervical lymph node metastasis before surgery in patients with cN0 SCC of the tongue with high (C-index = 0.846) and good calibration.

Specific features of our MRI-based nomogram include the use of four potential predictors of rTTT, PLD, tumor size and tumor location that is a set of continuous scale representation of three-dimensional evaluation. This comprehensive evaluation would increase the predictive power of the nomogram. Moreover, we could achieve the nomogram optimal cutoff value of 210 points with high sensitivity of 93.8% and specificity of 87.5% for classifying occult nodal metastasis risk in our study populations. Then we tested the predictive accuracy of the nomogram to identify patients at high risk for pre-operative, clinically undetectable cervical lymph node metastasis in the validation cohort. The reliable results showed that the nomogram had a good predictive ability with an AUC of 0.881, sensitivity of 83.3%, specificity of 79.3% and well-generalized calibration. This demonstrated a suitable degree of accuracy and quantified the ability of the nomogram to discriminate between patients with and without cervical lymph node involvement.

A predicted probability of metastasis rate > 20% was presented in patients with high metastasis risk group who obtained a total score from the nomogram of more than 210 points. Clinical T2 and tumor located in the posterior of tongue were commonly found in the high metastasis risk group. A study performed by Feng et al. [4] suggests that END should be a preferred treatment strategy for tongue carcinoma in stage T2. A study by Sagheb et al. [32] and Jiang et al. [30] reported that the probability of cervical metastases rate of tongue SCC located in the posterior third region was significantly higher than tumors located in the anterior two-third of the tongue. It is possible that the posterior area of the tongue’s supply of lymphatic drainage to the superficial and deep cervical lymph nodes creates a greater chance of developing positive neck node disease.

Cancer cells typically spread in different planes to invade surrounding structures, and evaluation in three-dimensions is a more accurate prediction, reflecting the entire tumor extension. Therefore, we selected the technique measurement similar to the study of Kwon [21] to investigate MRI-assessed tumor thickness and identified cutoff values in three planes. A logistic regression model showed that significant rTTT resulted in a more accurate metric to reflect the extent of cancer invasion and comprehensive interpretation of tumor characteristics than minimal rTTT. It was determined that MRI-assessed tumor thickness extending beyond the cutoff value in at least two directions significantly increased the nodal metastasis rate. The results showed that rTTT represents the better result of MRI-derived thickness and should be practically adopted in determining metastasis outcome.

Current reliable evidence suggests that the most common MRI-assessed tumor thickness cutoff values available range from 7.5 mm to 12.3 mm [11, 12, 16–18, 20, 21]. Therefore, it is difficult to compare between studies because previous studies have measured MRI-assessed tumor thicknesses from various techniques and varying planes. This study determined that the clinically useful cutoff value of MRI-assessed tumor thickness was 8.5 mm, 11 mm and 12 mm in axial, coronal and sagittal planes, respectively. The predictive power of aTT was 84.4% sensitivity, 86.4% specificity and sTT was 90.6% sensitivity, 81.8% specificity to detect cervical lymph node involvement (*P* < 0.001). This study reported cutoff value consistent with previous studies of MRI thickness in tongue and oral cancer [14–22]. As study by Hu et al. [20] reported MRI-assessed tumor thickness of 8.5 mm was useful in predicting the prognosis of lymph node metastases for 46 patients with tongue squamous cell carcinomas. Park et al. [18] studied 49 patients with tongue carcinoma and reported the relationship between MRI invasion depth and nodal metastasis with a cutoff value of 9.5 mm on T1WI. Jung et al. [17] reported on 50 patients with T1–T2 tongue carcinoma and recommended MRI invasion depth cutoff values of 11 mm and 12 mm for T1- and T2-weighted cases, respectively, and showed significant correlation with nodal metastasis. This was similar to results of Kwon et al. [21] that reported MRI-assessed tumor thickness cutoff value of 12.3 mm in the sagittal plane to be significantly different to evaluate cervical metastasis.

Recently, pDOI has become an essential predictor of occult nodal metastasis [31–35]. Several studies have emphasized the correlation between pathological thickness and MRI thickness [14–22]. The current study confirmed a strong correlation between MRI and pathological thickness [*r* = 0.836]. The eighth edition of the AJCC proposed incorporating pDOI into three groups (5 mm, from > 5 to 10 mm, and > 10 mm) [33]. This study found that none of the patients whose pDOI was less than 5 mm detected nodal metastasis, while all whose pDOI exceeded 5 mm the occult metastasis incidence rate was occurred > 20%. These results correspond with a previous studiy of a meta-analysis by Huang et al. [31] that reported a statistically significant difference between the 4 mm and 5 mm thickness cutoff values for cervical metastasis in oral carcinoma. Previous studies in tongue carcinoma also stated that higher incidence of nodal metastasis was detected when pDOI > 5 mm [2, 17, 34]. This is likely because the intrinsic muscle of the tongue is a poor barrier to tumor spread drainage to cervical lymphatics system.

Other MRI predictors describing tumor extension included SLD and PLD. SLD did not seem useful for prediction of the existence of lymph node metastasis. Multivariate analysis showed PLD with a cutoff value of 5 mm was a more reliable predictor of nodal metastasis than SLD, and this was included in the nomogram. Another valuable finding was that the risk of nodal metastasis was > 20% in patients with PLD ≤5 mm. This may be due to increased tumor extension to the lingual lymph nodes and lingual vessels on the lateral aspect of tongue may have a greater chance to develop metastasis. Lingual lymph node metastasis have been reported in oral squamous cell carcinoma [36]. Similar results were to reported in a study by Okura et al. [16] that reported that in 43 patients with SCCT, PLD was a significant predictor of lymph nodal metastases. END decision may be based on PLD < 5.2 mm. As previously mentioned, PLD was an outstanding predictor that may represent tumor extension and potentially has a role in predicting nodal metastasis.

This study has multiple limitations. First, the nomograms were constructed using retrospective data, which might introduce the risk of treatment selection bias. Also, sample size in our study was limited to the oral tongue sub-site and single center analysis. Therefore, these nomograms must be further validated in a larger prospective cohort in other sub-sites, and accurate risk estimates are required for the clinical trial design. A prospective study is ongoing to evaluate the effectiveness of the proposed nomogram and determine other potential predictive variables. The follow-up period in this study should have been longer to provide more accurate in predictions. Standardized technique and resolution of imaging to measure MRI thickness should be clarified and used to identify the precise cutoff value necessitating further study to confirm our findings.

## Conclusions

In summary, the nomogram demonstrated predictive accuracy of AUC 0.952 in the primary and AUC 0.881in the validation cohorts. The effect of three-dimensional evaluation of MRI predictors impacted the predictive accuracy of the preoperative nomogram. Assessment of occult metastasis risk value obtained from the MRI-based nomogram can assist in preoperative decision making of whether to perform neck surgery for individual patients with early-stage SCCLT. The probability of nodal metastasis tended to be greater than 20% in patients with high metastasis risk, or nomogram total score > 210 points, and the nomogram suggests that these patients should be treated with prophylactic neck dissection. It was concluded that the multiparametric MRI-based nomogram displayed reasonable accuracy and discrimination was generated and externally validated to predict individualized metastasis outcome.

## Data Availability

The datasets used and/or analysed during the current study are available from the corresponding author on reasonable request.

## Abbreviations

SCCLT: squamous cell carcinoma of lateral tongue
cN0: clinically node-negative
AJCC: American Joint Committee on Cancer
MRI: magnetic resonance imaging
3D: three dimensions
rTTT: radiologic tumor thickness threshold
aTT: axial tumor thickness
cTT: coronal tumor thickness
sTT: sagittal tumor thickness
PLD: paralingual distance
SLD: sublingual distance
pDOI: pathologic depth of invasion
pTT: pathologic tumor thickness
PNI: perineural invasion
LVI: lymphovascular invasion
ROC: receiver operating characteristic
AUC: area under the curve
PPV: positive predictive value
NPV: negative predictive value
LR +: positive likelihood ratio
LR −: negative likelihood ratio
OR: odds ratio
CI: confidence interval
pN +: positive neck-node
END: elective neck dissection
